# Formation of circular polyribosomes on eukaryotic mRNA without cap-structure and poly(A)-tail: a cryo electron tomography study

**DOI:** 10.1093/nar/gku599

**Published:** 2014-07-12

**Authors:** Zhanna A. Afonina, Alexander G. Myasnikov, Vladimir A. Shirokov, Bruno P. Klaholz, Alexander S. Spirin

**Affiliations:** 1 Institute of Protein Research, Russian Academy of Sciences, 142290 Pushchino, Moscow Region, Russia; 2 Centre for Integrative Biology (CBI), Department of Integrated Structural Biology, IGBMC (Institute of Genetics and of Molecular and Cellular Biology), Centre National de la Recherche Scientifique (CNRS) UMR 7104 / Institut National de la Santé de la Recherche Médicale (INSERM) U964 / Université de Strasbourg, 1 rue Laurent Fries, 67404 Illkirch, France.

## Abstract

The polyribosomes newly formed on recombinant GFP-encoding mRNAs in a wheat germ cell-free translation system were analyzed using cryo-electron tomography, with sub-tomogram averaging of polysomal ribosomes and reconstruction of 3D structures of individual polyribosomes. The achieved level of resolution in the reconstructed polyribosomes allowed deducing the mRNA path by connecting adjacent exit and entry sites at the ribosomes inside each polyribosome. In this way, the circularity of a significant fraction (about 50%) of translating polyribosomes was proved in the case of the capped poly(A)-tailed mRNA, in agreement with the existing paradigm of the circularization via interaction of cap-bound initiation factor eIF4F with poly(A)-binding protein. However, translation of the capped mRNA construct without poly(A) tail, but with unspecific 3′-UTR derived from non-coding plasmid sequence, also led to the formation of circular polyribosomes in similar proportion (40%). Moreover, the polyribosomes formed on the uncapped non-polyadenylated mRNA with non-synergistic 5′- and 3′-UTRs proved to be circular as well, and appeared in the same proportion as in the previous cases. Thus, the formation of circular polyribosomes was found to be virtually independent of the presence of cap structure and poly(A) tail in mRNA, in contrast to the longstanding paradigm in the field.

## INTRODUCTION

Polyribosomes were discovered at the beginning of the 1960s as the main form of organization of translating ribosomes (1–6). The arrangement of membrane-bound ribosomes into specific clusters was described even earlier (7). The polyribosome is defined as a group of ribosomes strung on a messenger ribonucleic acid (mRNA) chain and translating it sequentially in one direction, from the 5′ terminus to the 3′ terminus. A number of early electron microscopy observations demonstrated the circular array of ribosomes in eukaryotic polyribosomes (8–10) [see also later publication, (11,12), for membrane-bound circular polyribosomes]. The observations of circular forms of eukaryotic polyribosomes were consistent with the experiments providing evidence for circular translation of mRNA when ribosomes moving along the mRNA chain were not released after termination, supposedly reinitiating a new round of translation on the same mRNA chain (13–15). Later, numerous experiments revealed functional synergism, as well as some physical interactions between the cap structure and cap-dependent initiation factors, on the one hand, and poly(A) tail and poly(A)-binding protein (PABP), on the other hand (16–22). The capability of the cap-binding initiation factor eIF4F located at the capped 5′-end of mRNA to physically interact with the 3′-located PABP and the resultant circularization of isolated mRNA chain under *in vitro* conditions were experimentally demonstrated (23). Those results were in agreement with the ‘closed-loop model’ that implied the initiation-coupled circularization of capped polyadenylated mRNAs [(24); see also (25)]. Since then the model of cyclic polyribosome formation on the eukaryotic mRNAs by circularization due to joining of their 5′ and 3′ regions via the protein-to-protein (eIF4F-PABP) bridges was widely recognized and became generally accepted. However, neither direct experimental proof of indispensable participation of cap-structure and poly(A)-tail in the eukaryotic polyribosome circularization has been obtained, nor electron microscopy (EM) analyses of polyribosomes formed on uncapped and/or poly(A)-tailless mRNAs, in comparison with those formed on capped polyadenylated mRNAs, have been done.

Meanwhile, in search of efficient mRNA constructs for practical use in long-term cell-free translation systems (26–28), several translation-enhancing non-capped 5′-UTRs (untranslated regions) were found. Among them, the 61-nt leader sequence of mRNA encoding for a light-emitting protein of hydroid polyp *Obelia longissima*, obelin, was shown to provide a fairly good level of cap-independent translation of heterologous mRNAs in a cell-free translation system based on the wheat germ extract (29). The electron microscopy study of negatively stained polyribosomes formed on the uncapped non-polyadenylated mRNAs with this leader sequence (here designated as 5′-UTR_Obe_) demonstrated predominantly double-row arrangement of ribosomes that was interpreted in terms of circular topology of polysomal mRNA (‘collapsed circles’, see the Discussion section) (30). This interpretation was confirmed by functional tests that provided evidence for efficient re-initiation of terminating polysomal ribosomes at the same mRNA (30). Thus, it became necessary to resolve the conflict between the above-mentioned results and the widely accepted model of eukaryotic mRNA circularization via cap–poly(A) interaction. The question arises: are the polyribosomes formed on an uncapped mRNA indeed circular? If yes, the cap-structure and poly(A)-tail cannot be considered as necessary attributes of the circular polyribosomes, and the paradigm should be changed.

To answer the question we used the methodology of cryo electron microscopy where polyribosomes were fixed in amorphous ice by flash-freezing without being adsorbed on a support surface thus avoiding structural deformations that could result from interactions with the surface, as well as from staining and drying procedures. In this work, we prepared the uncapped mRNA construct composed from 5′-UTR of obelin mRNA, the coding region of GFP mRNA, and the 3′-UTR of TMV RNA. Using the technique of cryo electron tomography (cryo-ET), we demonstrate the formation of circular polyribosomes on this uncapped mRNA without poly(A) tail in a wheat germ cell-free translation system. No differences were found in forms and proportions of circular polyribosomes in the cases of the uncapped mRNA without poly(A) tail and the capped mRNA composed of the 5′-UTR of β-globin mRNA, GFP-encoding region and poly(A) tail. The circular polyribosomes were also observed when poly(A) tail in the capped mRNA was replaced with a non-specific plasmid sequence. Together, these results provide insights into cap- and poly(A)tail-independent polysome circularization that calls for a revision of the longstanding paradigm in the field.

## MATERIALS AND METHODS

### Preparation of mRNAs

Capped mRNA with poly(A) tail, consisting of 5′-UTR of β-globin mRNA, N-tagged GFP-encoding sequence (975 nt) and polyadenylated 3′-UTR (designated *Cap-5′UTR_βGlobin_-scGFP-3′UTR_(N)40-(A)100_*), was prepared with the use of pTZβG-SBP-CBP-GFP-A30 plasmid (31), containing the following sequences under the T7 promoter: 5′-UTR of rabbit β-globin mRNA, coding sequence of GFP (‘cycle3’ mutant) fused at its N-end with SBP-CBP tag (encoding for streptavidin- and calmodulin-binding peptides) and at the C-end with His_6_ tag, and 3′-terminal A_30_ sequence. The plasmid was linearized with BglII (A_30_ sequence was cut off in this way, leaving 40-nt spacer after stop codon), and capped mRNA was prepared by *in vitro* transcription with T7 polymerase (Fermentas), as described previously (32), with the use of the oriented cap-analog 3′-O-Me-m^7^GpppG (New England Biolabs). Poly(A)-tail to the mRNA was added in reaction with poly(A) polymerase (Epicentre). The polyadenylation mixture containing 0.6 mg/ml of mRNA, 1 mM ATP, 1 U/μl of RNase inhibitor (Fermentas), 0.08 U/μl of poly(A) polymerase in 50 mM Трис-HCl pH 8.0, 250 mM NaCl and 10 mM MgCl_2_ was incubated for 30 min to add poly(A) of about 100 nt in length. The length of synthesized poly(A) tail was controlled by RNase H (Thermo Scientific) hydrolysis with DNA oligonucleotide complementary to spacer region followed by urea-polyacrylamide gel electrophoresis analysis.

Capped mRNA without poly(A) tail, consisting of 5′-UTR of β-globin mRNA, N-tagged GFP-encoding sequence (975 nt) and a non-specific 180 nt 3′-UTR derived from plasmid non-coding sequence (designated *Cap-5′UTR_βGlobin_-scGFP-3′UTR_(N)180_*) was prepared with the use of pTZβG-SBP-*CBP*-GFP-ΔUTR plasmid, derived from pTZβG-SBP-CBP-GFP-A30 by excision of A_30_-containing fragment between BglII and XbaI sites and ligation of blunted ends (the absence of A_30_ was verified by restriction analysis). The plasmid was linearized with PvuI, and the capped mRNA was prepared in the same way as the above-described mRNA, but without polyadenylation.

Uncapped non-polyadenylated mRNA (*5′UTR_Obe_-GFP-3′UTR_TMV_*) was prepared by *in vitro* T7-polymerase transcription of pObe-GFP-TMV plasmid linearized with EcoRI. The plasmid was constructed on the basis of pUC19 vector by inserting between HindIII and EcoRI sites the fragment containing 5′-UTR of obelin mRNA, GFP-encoding sequence (750 nt) and 3′-UTR of tobacco mosaic virus (TMV) RNA. The fragment was prepared in 3-primer polymerase chain reaction with pYGFP plasmid (kindly provided by Y. Endo, Ehime University, Japan). The primers T7-Obe (5′TGCCAAGCTTAATACGACTCACTATAGATCTAA CCAAACAACTCAGCTCACAGCTACTGAACAAC), Obe-GFP (5′GCTCACAGCTACTGAACAACTCTTGTTGTGTACAATCACCATGACTAGCAAAGGAGAAGAAC) and TMV-r (5′CAGTGAATTCCGCATATATGGGC) were used in the 20:1:20 ratio. The sequence of 5′-UTR of obelin mRNA was introduced in primers T7-Obe and Obe-GFP.

The *5′UTR_Obe_-GFP-3′UTR_TMV_* mRNA modified at the 3′-end with fluorescein-5-thiosemicarbazide (Invitrogen) was prepared as described in (31).

### Cell-free translation

Translation of the mRNAs was performed in a continuous exchange cell-free system (CECF) based on wheat germ extract provided by Roche Diagnostics, Penzberg, Germany, or prepared in our laboratory using the similar protocol (28). The polyribosomes formed during the first 15 min of translation were analyzed. Translation mixture contained 25 mM HEPES-KOH рН 7.6, 2.0 mM Mg(OАc)_2_, 85 mM KOAc, 2 mM dithiothreitol, 0.25 mM spermidine, 2% glycerol, 0.01% NP40 detergent, 0.03% NaN_3_, 1 mM ATP, 0.4 mM GTP, 0.3 mM each of 20 amino acids, 0.05 mg/ml of total yeast tRNA (Roche Diagnostics), 16 mM creatine phosphate (Roche Diagnostics), 0.1 mg/ml of creatine phosphokinase (Roche Diagnostics), 500 U/ml of RNase inhibitor (Ambion), 30% (v/v) of wheat germ extract, and optimal concentration of mRNA (100 nM, 200 nM or 300 nM, as indicated in Supplementary Figure S1). Feeding solution contained the same components, except tRNA, creatine phosphokinase, RNase inhibitor, wheat germ extract and mRNA. Translation mixture was pre-incubated for 2 min at 25°C prior to the addition of mRNA. Hundred microliters of translation mixture were placed into the reaction chamber of the CECF reactor; the outer chamber received 1 ml of feeding solution. The reactor was incubated at 25°C with agitation of feeding solution. To monitor the translation reaction, 3 μl aliquots were taken at specific time points and quenched by addition of cycloheximide (0.01 mg/ml); GFP fluorescence (excitation 395 nm, emission 510 nm) was measured after overnight incubation on ice. Polyribosome profiles in the cell-free translation mixtures were analyzed by sedimentation in 15–50% sucrose gradient as described previously (30) (see Supplementary Figure S2). About one third of ribosomes in the translation mixture were found in polyribosome fractions.

### Cryo electron tomography

Samples for cryo-ET analysis were taken after 15 min of translation, and immediately diluted 5-fold with ice-cold buffer A (25 mM HEPES-KOH pH 7.6, 0.5 mM Mg(OAc)_2_, 85 mM KOAc, 0.01 mg/ml cycloheximide). Colloidal gold markers (10-nm gold•protein A conjugate, purchased from the Department of Cell Biology, Utrecht University, The Netherlands) were added to give a final distribution of 10–20 beads per EM field. Within 1 min after dilution, 3 μl of the sample was applied to 300 mesh holey carbon grids (Quantifoil 2 × 2) in the Vitrobot apparatus (FEI, Eindhoven, The Netherlands) at 20°C, 95% humidity. Excess sample volume was blotted automatically with filter paper from both sides of the grid, and it was immediately plunged into liquid ethane pre-cooled in liquid nitrogen.

Cryo-ET data were recorded on Polara F30 and Titan Krios FEG instruments (FEI, Eindhoven) run at 150 kV or 300 kV acceleration voltage at magnification 23 000 or 31 000 and a nominal underfocus of Δ*z* = −3 μm. Tilt series were recorded between −66° and +66° with a tilt angle increment of 1°–3° and with a cumulative electron dose not exceeding 60 e^−^/Å^2^. Data acquisition was carried out using a 4096 × 4096 CCD Eagle or CMOS Falcon I camera (FEI, Eindhoven), or a back-thinned Falcon II camera for the Titan Krios data. The image alignment and tomogram reconstructions were performed by using the ETOMO (IMOD software, Boulder Laboratory for 3D EM, University of Colorado, CO, USA) and inspect3D (FEI, Eindhoven) software. Sub-tomograms of the individual polysomal ribosomes images were extracted and subjected to maximum likelyhood based iterative alignment and averaging for each tomogram using Xmipp software package (Spanish National Center of Biotechnology, Madrid) (33). Sub-tomogram averages were calculated for each individual tomogram separately based on cryo-ET data only, no external (reference) model was used in averaging. The contour level (threshold) of the sub-tomogram average was adjusted accordingly, to get the best fit with the cryo-EM structure of wheat ribosome [EMD-1125, (34)] filtered to 20 Å resolution. The adjustment was done in UCSF Chimera software (35). Positioning of the sub-tomogram averages back into the tomograms was done using Xmipp and Chimera softwares. In total, 15 tomograms were recorded and 424 polyribosomes containing 2332 ribosomes were analyzed by sub-tomogram averaging. From 130 to 160 polyribosomes were reconstructed for each mRNA used, with estimated resolution of the averaged ribosome structure of 4–5 nm.

## RESULTS

### Averaged ribosome structure, polyribosome reconstruction and mRNA path tracing

In the averaging process, the maximum-likelihood analysis of a full set of polysomal ribosomes of the tomogram (sub-tomograms) was performed, accompanied by an orientation survey for each ribosome: during sub-tomogram averaging process Xmipp software rotates and probes each polysomal ribosome for best alignment with all other polysomal ribosomes of the tomogram; in this way the averaged ribosome map for the tomogram is obtained, and at the same time the initial orientation of each ribosome is determined from the record of its rotation.

**Figure 1.**
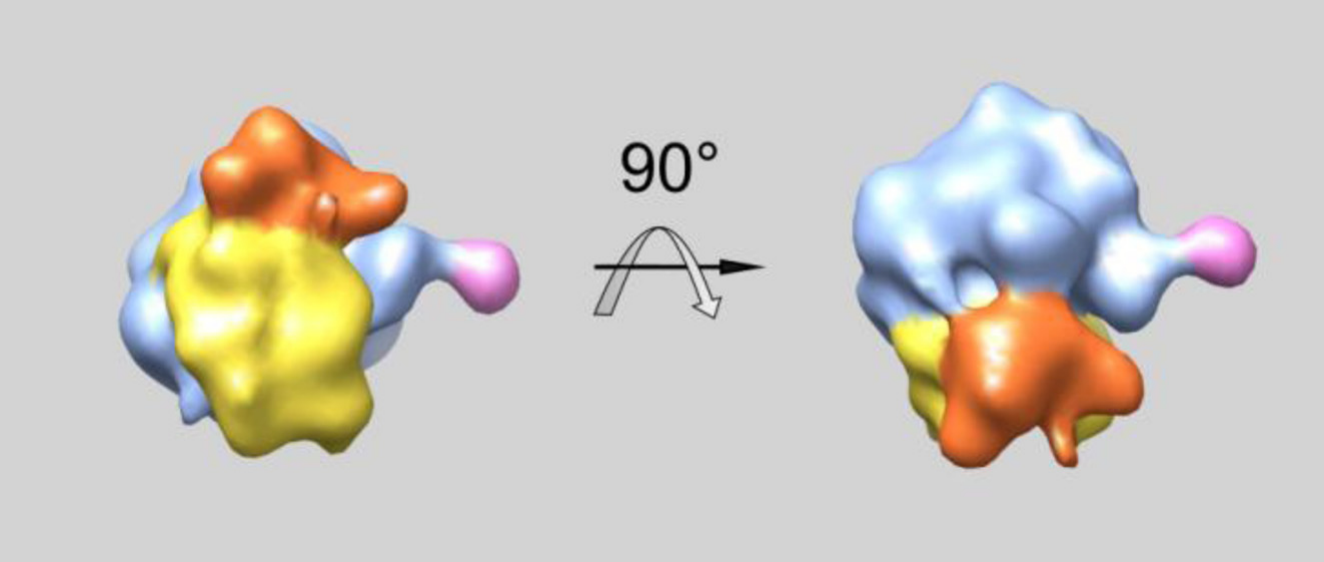
Ribosomal model obtained by sub-tomogram averaging. The head of the 40S subunit is shown in red, the body of the 40S subunit in yellow, the P1/P2 stalk of the 60S subunit in pink and the 60S subunit in blue.

This analysis yielded the averaged ribosome structure (Figure 1) and the 3D map of all the polysomal ribosomes within the tomogram.

The major characteristic structural features of the ribosome, the P1/P2 stalk of the 60S subunit and the head of the 40S subunit with its beak are well seen in the reconstructed structure. As no external template was used in averaging, this result confirms the correctness of the structure and, most important, the correctness of the orientation angles that were determined for each polysomal ribosome within the tomogram. The averaged ribosome structure was then placed back to the tomogram in positions and spatial orientations determined for each polysomal ribosome during averaging process. In this way the reconstructed tomograms were obtained and the reconstructed structures of individual polyribosomes were reconstituted.

Next, the orientation of ribosomes in each polysome was analyzed to determine the mRNA route. As the mRNA path inside the ribosome is well known (36,37), the direction of the mRNA chain through the polysomal ribosomes can be assigned and therefore the overall path of the mRNA chain within each polyribosome can be inferred. In most polyribosomes the unique continuous route could be found, in which the mRNA chain connects the neighboring ribosomes without kinks or knots. Thus, the mRNA paths in all polyribosomes of the reconstructed tomograms were traced, and the polyribosomes could be classified according to topology of their mRNA. A polyribosome was recognized circular, when the inferred mRNA path was arranged in a continuous closed loop, either as a circle within a ring-shaped polyribosome or as two anti-parallel strands within a double-row polyribosome with mRNA ends come together at the same flank (31). A double-row polyribosome was qualified linear (i.e. non-circular in topological sense), when the mRNA path passed in a zigzag-like manner from one end of a double-row to the other end, thus the mRNA 5′ and 3′ ends being apart at opposite flanks. Polyribosomes with single-row or 3D-helical arrangements of ribosomes were annotated linear. Tetrasomes were considered circular if appeared square-shape with continuous mRNA path. Shorter forms (dimers and trimers) were not assigned in this classification, as dimerization is known to occur without mRNA and translation, and trisomes cannot provide reliable unambiguous tracing of mRNA pathway. It should be noted that in the present work the circular topology of mRNA path has been assigned only after analysis of the polysome in 3D tomographic image. In some cases the arrangement of ribosomes in the polysome looking ring-shape in projection did not confirm the continuous mRNA path through the circle, and therefore it was considered non-circular (linear).

### Polyribosomes formed on capped mRNA with poly(A) tail

**Figure 2.**
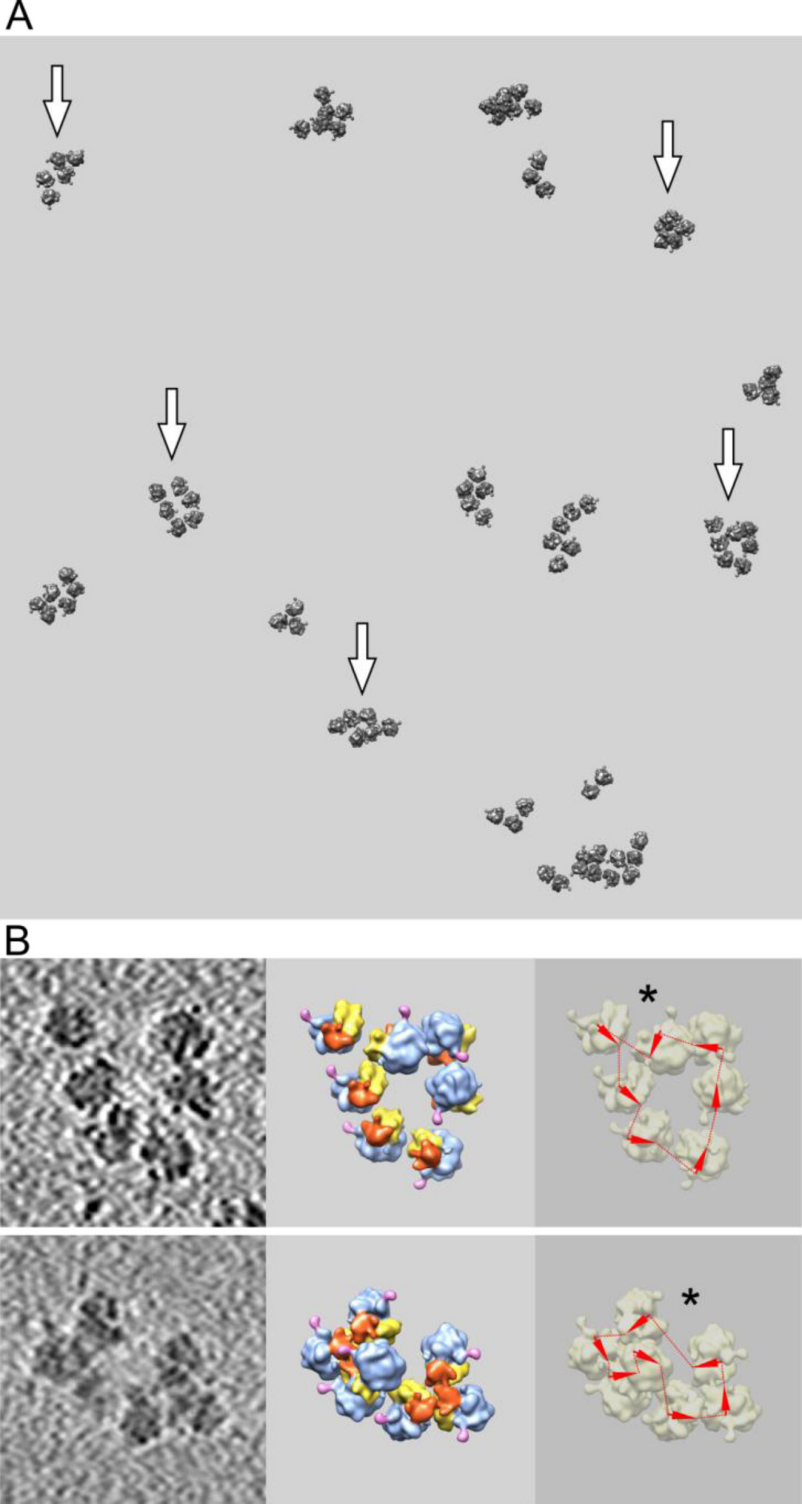
Cryo-ET analysis of polyribosomes formed in the wheat germ cell-free system with capped polyadenylated mRNA (*Cap-5′UTR_βGlobin_-scGFP-3′UTR_(N)40-(A)100_*) after 15 min of translation. (**A**) Fragment of reconstructed tomogram. Polyribosome models were obtained by fitting the averaged ribosome model as described in text and the Materials and Methods section (monoribosomes are not shown). Arrows point to polyribosomes with confirmed circular mRNA configuration, either ring-shape or double-row polyribosomes. (**B**) Left: tomographic slices of individual polyribosomes*;* middle: 3D subtomogram reconstructions of the same polyribosomes. Color indications are the same as in Figure 1. Right: deduced mRNA path within polyribosomes. Orientation of the mRNA chain in each ribosome is symbolized by an arrowhead directed from the mRNA entry to mRNA exit sites of the ribosome. The deduced path of the mRNA chain through the ribosome is shown by a line. The asterisk points to putative 3′-5′ junction.

Figure 2 shows the results of cryo-ET analysis of the polyribosomes formed on the capped mRNA with poly(A) tail. Panel A presents one of the typical reconstructed tomograms with polyribosomes formed in the translation system for 15 min. During this time the polyribosomes attain their normal size, consisting mainly of five to seven ribosomes (pentasomes, hexasomes and heptasomes). Tetrasomes may also be seen on tomograms, along with disomes (or dimers) and trisomes. Although ideal ring-shaped polyribosomes were not often encountered, the circularity of a significant number of polyribosomes (about 50% of all polysomal population; see Supplementary Data, Supplementary Table S1) was revealed by the analysis of mRNA topology in all polyribosomes of the relevant tomograms. The analysis was based on finding of mutual ribosome orientations and tracing of mRNA path, as described above [see also (31)]. The polyribosomes with confirmed topological circularity are indicated by arrows in the following figures.

In Figure 2B, two examples of tomographic slices of individual circular polyribosomes and their 3D structures derived from tomographic averaging of polysomal ribosomes followed by polyribosome reconstruction are presented. Here and in the next figures of this paper the ‘left-hand frames’ show slices of the original tomogram fragments, each with one selected polyribosome. The ‘middle frames’ expose 3D reconstructed structures of the selected polyribosomes with colored characteristic features of their ribosomes (see Figure 1). The ‘right-hand frames’ show the result of mRNA path tracing based on the mutual orientation of the mRNA exit and enter sites of ribosomes in polyribosomes.

In the right-hand frames (Figure 2B) the mRNA path in each ribosome is indicated by an arrowhead (3′–5′ direction), whereas the dashed line connects the mRNA exit and entry sites of neighbor ribosomes in the polyribosome. From the orientation of P1/P2 stalk it is seen that most ribosomes are regularly arranged within the polyribosome circle, with their P1/P2 stalks pointing in the same direction along the circle; the 40S subunits are usually facing inside the circle, and their heads are apt to be exposed on the same side of the ring. With this arrangement the mRNA chain can successively pass from the mRNA exit to the mRNA entry sites of the 5′-adjacent ribosomes, so that the overall circular mRNA pathway is defined. Some ribosomes, however, may deviate from the polyribosome ring plane, but still keeping the P1/P2 orientation and, hence, the direction of the mRNA path; in such a case they may not be seen in the plane of a tomogram slice shown in the left-hand frames, but become visualized upon 3D reconstruction of the polyribosome (see Supplementary Figures S6 and S7). Such a situation is observed in both examples presented in Figure 2B. The polyribosome illustrated in the upper row of frames consists of seven ribosomes, as follows from the reconstructed polyribosome image (middle frame), but one ribosome is located outside the selected tomographic slice (left-side frame). The other example of topologically circular polyribosome given in the lower row of frames is similar: one ribosome of the heptasome, supposedly abutted on the 3′-5′-end junction, is bulged from the ring plane (see middle and right-side frames), so that the polyribosome looks like a hexasome at the tomogram slice (left-side frame).

The tendency to form antiparallel juxtapositions of two halves of the topologically circular polyribosomes deserves attention. Two antiparallel rows of ribosomes are seen in hexasomes marked by arrows, as well as in the marked pentasome and heptasome in Figure 2A. This tendency seems to be related to the problem of the so-called double-row polyribosomes that will be specially considered in the Discussion section.

### Polyribosomes formed on capped mRNA without poly(A) tail

**Figure 3.**
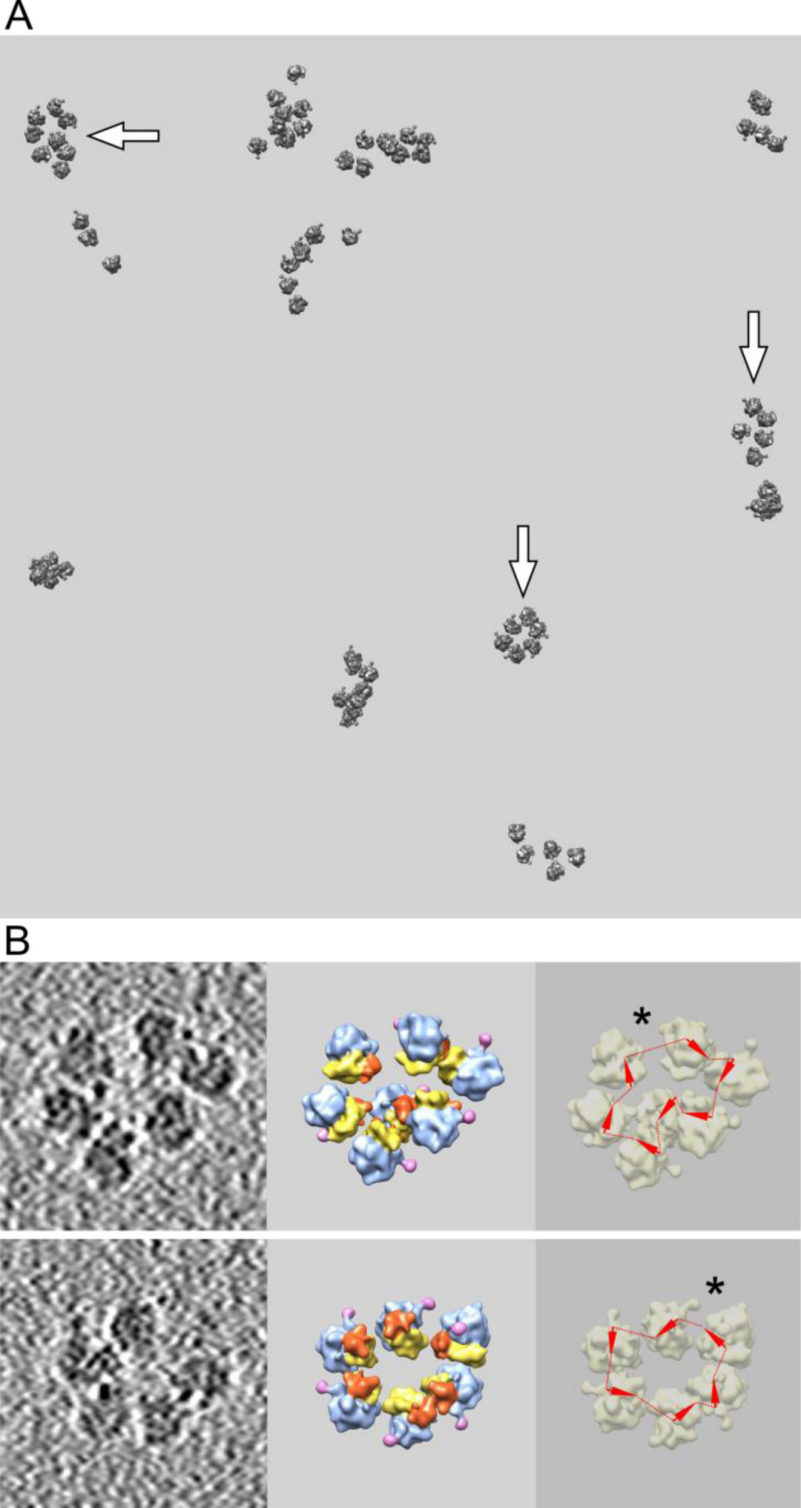
Cryo-ET analysis of polyribosomes formed in the wheat germ cell-free system with capped mRNA without polyA (*Cap-5′UTR_βGlobin_-scGFP-3′UTR_(N)180_*) after 15 min of translation. (**A, B**): see the legend in Figure 2.

The results of cryo-ET study of polyribosomes formed on the capped mRNA with the same 5′-UTR and coding sequence as above, but without poly(A) tail and with a non-specific 180 nt sequence as 3′-UTR instead, are presented in Figure 3. Panel A is the slice of a reconstructed tomogram with polyribosomes assembled during 15-min translation. As compared with the polyribosomes formed on the capped polyadenylated mRNA (Figure 2), the polyribosomes devoid of poly(A) display no principal differences. Despite the absence of a poly(A) tail, the circular polyribosomes are formed and present in a significant proportion (about 40% of all polysomal ribosomes, which compared well with the 50% observed for polysomes containing poly(A)-tail mRNA). In the field of images presented in Figure 3A, well recognizable circular pentasome, hexasome and octasome are seen; their circularity was checked by the ribosome orientation and mRNA tracing analysis (they are marked by arrows). Thus, the absence of poly(A) tail exerted no drastic effect on the formation of circular polyribosomes. It should be mentioned that the translational activity of the cell-free system with the mRNA without poly(A) was also comparable to that with the capped polyadenylated mRNA, on condition that the mRNA concentrations optimal for each mRNA were used (the optimal translation activity of the construct without poly(A) tail required two times higher concentration than the optimum for capped polyadenylated mRNA; see Supplementary Figure S1). Sedimentation profiles of polyribosomes formed on both mRNAs were also similar (Supplementary Figure S2).

Figure 3B (see also Supplementary Figures S8 and S9) shows two examples of circular polyribosomes. One of the examples (the upper row of frames) looks like an ideal ring-shaped hexasome in the tomographic slice, but in reality it is a heptasome: the seventh ribosome is placed above the tomographic slice plane (compare the left and middle frames). The other example (the lower row of frames) is an almost ideal ring-shaped hexasome, but with one ribosomal particle being slightly outside the plane of the tomographic slice and thus invisible in the left frame. As in Figure 2, the asterisks in the right-side frames indicate the irregularities in the ring-shaped polyribosomes that may be the sites of the 3′-5′ junctions of polysomal mRNA.

### Polyribosomes formed on uncapped mRNA without poly(A) tail

**Figure 4.**
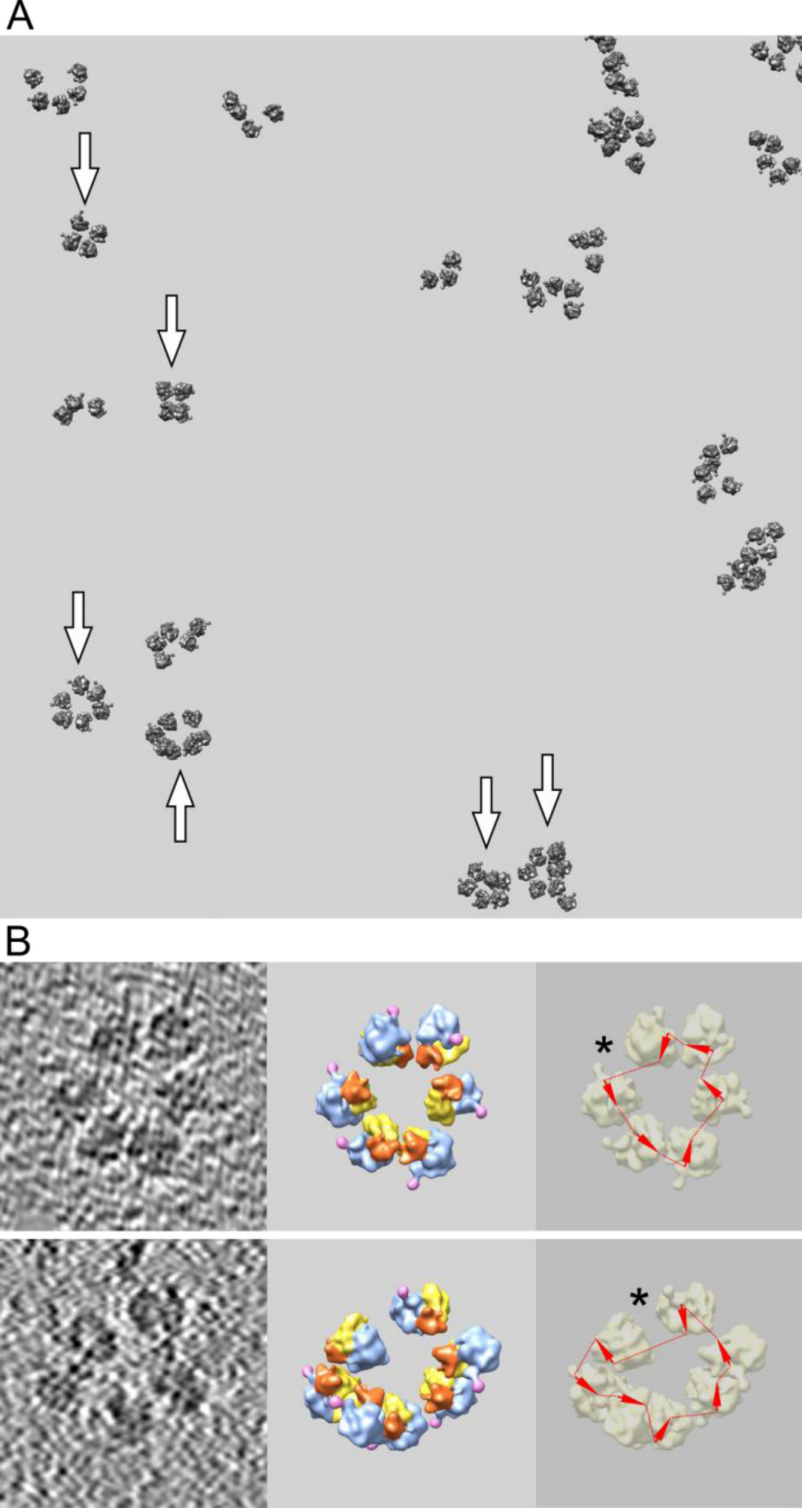
Cryo-ET analysis of polyribosomes formed in the wheat germ cell-free system with uncapped non-polyadenylated mRNA (*5′UTR_Obe_-GFP-3′UTR_TMV_*) after 15 min of translation. (**A, B**): see the legend in Figure 2.

The most impressive result was obtained with polyribosomes formed on the mRNA construct devoid of both cap structure and poly(A) tail. As already mentioned, it was composed from the uncapped 5′-UTR of obelin-encoding mRNA, the coding region of GFP mRNA and the 3′-UTR of TMV RNA. It should be emphasized that our choice of this 5′-UTR–3′-UTR pair provided for absence of any phylogenetic or ecological relationship between their biological sources (hydrozoa and plant virus), so that the possibility of a specific affinity of the UTRs for each other is unlikely. Figure 4 shows that the polyribosomes formed on the uncapped poly(A)-less mRNA with these highly heterologous UTRs were also found in the circular, often true ring-shaped configuration. According to the statistics, the fraction of circular polyribosomes, with the circularity confirmed by the analysis of ribosome orientation and mRNA path, approached 40% of the total polysomal ribosomes, as in the previous case (see Supplementary Table S1).

In Figure 4A, a tomographic field with reconstructed polyribosomes formed on the uncapped poly(A)-less mRNA is shown, and the confirmed circular polyribosomes are marked by arrows. Ring-shaped tetrasomes, pentasomes and hexasomes formed during 15 min translation are present. Translation activity of the system with this mRNA construct was found even somewhat higher than that with the capped polyadenylated mRNA at optimal concentrations of both mRNAs, though the optimum concentration for uncapped non-polyadenylated mRNA was higher than that of the capped polyadenylated construct (see Supplementary Figure S1).

Figure 4B (see also Supplementary Figures S10 and S11) demonstrates two examples of circular polyribosomes, one being a ring-shaped hexasome (upper frames) and the other representing a heptasome with one ribosome bulging out from the tomographic slice plane. The asterisks in the right-side frames mark the irregularities in the ring-shaped polyribosomes that may be considered as the sites where the 3′-and 5′-ends of the mRNA meet.

### Test for authenticity of the uncapped poly(A)-less polyribosomes

The puromycin reaction seems to be one of the most adequate tests for authenticity of the polyribosomes. The molecules of puromycin react with nascent peptides inside the ribosomes resulting in the release of the peptidyl-puromycin from the ribosomes, followed by dissociation of polyribosomes into mRNA and individual ribosomes. In our test experiments, the puromycin was added to concentration of 1 mM in the translation mixture after 15 min of translation, when circular polyribosomes were already formed. Under these conditions the preformed polyribosomes disappeared, and only monosomes and some amount di- and trisomes could be visible in cryo-EM images (see Supplementary Figure S3). The sedimentation analysis in sucrose gradient also showed the disappearance of polyribosomal peaks as a result of the puromycin treatment (see Supplementary Figure S4).

In addition, the presence of the mRNA in polyribosomes was demonstrated by sedimentation analysis using the mRNA labeled with fluorescein at 3′-end (see Supplementary Figure S5). At the start of incubation neither polyribosomes nor the mRNA was detected in the polyribosome zone, while after 15 min of translation the mRNA fluorescence was displayed in the polysome peaks. Thus, the result of the puromycin test and the fluorescent mRNA demonstrated the typical response of active polyribosomes to puromycin impact. It is evident from the tests that the groups of ribosomes were not just accidental aggregates of ribosomal particles, but functional ensembles of interdependent protein-synthesizing units and that a coding mRNA chain was present there and held the particles together. In other words, this demonstrates that the circular formations comprising ribosomes and coding RNA, even without cap structure and devoid of poly(A)-tail, are true functional polyribosomes.

## DISCUSSION

Although circular eukaryotic polyribosomes were observed by classical electron microscopy (using shadow casting or negative staining techniques) and described in several early reports (see references in the Introduction section), the cryo-ET methodology exploited in this work allowed us to resolve both the mutual orientation of polysomal ribosomes and the localizations of mRNA entry and exit sites of individual polysomal ribosomes in the native (flash-frozen) state. This provided the possibility to deduce the mRNA pathway through all ribosomes along each polyribosome under investigation. In such a way the topologies of polysomal mRNAs could be determined. This approach was used to reveal the polyribosomes with circular topology of their mRNA, as well as to verify the circular topology of the polyribosomes in cases of doubt, including those with configurations deviating from the ideal ring-shaped form.

It is noteworthy that no specific tight contacts were observed between ribosomes in the circular polyribosomes studied here, in agreement with a relatively low occupancy of mRNA coding sequence by translating ribosomes, not less than 100 nucleotides per ribosome, at this juvenile stage of polyribosome formation (15 min incubation). Indeed, heptasomes and octasomes in our experiments had the occupancy of about 100–140 nucleotides per ribosome. At the same time, despite the absence of direct inter-ribosomal contacts, the circular polyribosomes, as already mentioned in the Results section, display certain orderliness in the arrangement and orientation of their ribosomes. The mRNA seems to be the sole structural constituent holding the ribosomes of a polysomal circle together and dictating their predominant orientation within the circle, particularly the 40S subunits positioning inside the circle. On the other hand, it should not be forgotten that eukaryotic polyribosomes may be associated with a great number of concomitant proteins (38,39), whose contribution to polyribosome structure is still unknown.

The cryo-ET methodology used in this work also made it possible to look into the problem of the so-called double-row polyribosomes. Earlier studies employing classical electron microscopy using a number of unavoidable special procedures, such as adsorption on supporting film, negative or positive staining, drying, shadow casting, etc., often demonstrated the polysomal configuration that looked like two rows of ribosomes. Such a double-row polyribosome was interpreted as a collapsed polysomal circle, the two halves of which were tightly adjacent to each other. Such a double-row configuration was considered typical of circular polyribosomes formed on long mRNAs [see, e.g. (9,12,30,40–41)].

Meanwhile, it was reported that helical polyribosomes with linear topology of mRNA, rather than circular polyribosomes, were observed using the cryo-ET analysis of cultivated human cells (42). The authors claimed that their analysis of native-vitrified samples challenges the previous studies based on conventional EM of stained and surface-adsorbed samples (30,41), in which the double-row arrangement of eukaryotic ribosomes was interpreted as polyribosomes with circular mRNA topology. In response to the above-mentioned comment (42), a special analysis of the topologies of mRNA chain in mature double-row-like polyribosomes formed during 1 h in a cell-free system was performed (31). Both ends of polysomal mRNAs in the double-row polyribosomes were marked, with gold nanoparticles at the 3′-end and with initiating 40S ribosomal subunits at the 5′-end. The antiparallel arrangement of the two rows was demonstrated by EM observations of both labels at the same end of a double-row polyribosome. At the same time a certain part of the double-row polysomes was found with the labels at opposite ends. The tracing of the mRNA path by the cryo-ET analysis further confirmed that among the double-row-like polysomes both topological types—with circular topology of polysomal mRNA and with linear topology—were present, the linear topology being more prevalent among mature polyribosomes (31).

Thus, the following conclusion can be made from the above. Ring-shaped polyribosomes may collapse into double-row structures in the process of preparation of their samples for electron microscopy studies. This phenomenon is especially typical for big polyribosomes formed on long mRNAs [see, e.g. (9,30,40–41); see also (12) for membrane-bound polyribosomes]. It is evident that two halves of the polyribosome cycle become stuck together and thus form two antiparallel rows of mRNA-bound ribosomes. Such double-row polyribosomes retain their circular topology, as recently shown by the analysis of mRNA pathways in cryo electron tomographic images of the polyribosomes of this type (31). At the same time, helical and zigzag-like polyribosomes with linear topology of mRNA also appear during translation, being characteristic of later stages of the translation process. They may arise both from the fraction of initially linear polyribosomes and as a result of opening the circular polyribosomes (unpublished observations). Therefore, as classical (conventional) EM images of the helical and zigzag-like polyribosomes look like double rows, EM cannot reliably distinguish between these and true circular polyribosomes without performing a full reconstruction using cryo-ET.

Returning to the problem of circularity, a question remains regarding the structural constituents that close the cycle. The idea of a protein bridge between 3′ and 5′ ends of mRNA seemed to be the most realistic. However, as the paradigmatic cap•eIF4F•PABP•poly(A) connection at the 3′–5′-end junction region may be found only auxiliary or insufficient, it could be reasonable to investigate in more detail all possible physical and functional relations between the key multisubunit (and multifunctional) initiation factors, such as eIF3 and eIF4, on the one hand, and all the complex of termination machinery proteins, on the other hand. In any case, it must be admitted that the mechanism of non-covalent closure of the polyribosome ring still remains unsolved. Taking into account the experiments providing evidence for reinitiation of terminating ribosomes at the same mRNA (13–15,24,30), it is likely that this problem is closely related to the problem of the interactions between the eukaryotic termination machinery and the initiation factors (43). There is evidence in favor of the idea that the mechanisms of reinitiation during cyclic translation of mRNA (30) are relevant to those of the post-termination reinitiation on linear mRNA (43).

Thus, we conclude that the circularization of eukaryotic polyribosomes does not strictly require the presence of cap structure and poly(A) tail in mRNA. Nevertheless, one possibility could be that the circularization of eukaryotic polyribosomes is caused mainly by interactions of initiation factors with the termination machinery. There are indirect indications that the circularization is accompanied by reinitiation of translation escaping the ATP-dependent scanning of 5′-UTR (30). Such a reinitiation may proceed via post-termination ATP-independent diffusional (bi-directional) scanning, as recently proposed for reinitiation at a downstream open reading frame of eukaryotic bicistronic mRNAs (43). Indeed, the possibility of the eIF4F/ATP-independent scanning of a non-coding leader sequence has been recently demonstrated in the case of an internal landing of ribosomal particles at the 5′-UTR region (44).

## SUPPLEMENTARY DATA

Supplementary Data are available at NAR Online.

SUPPLEMENTARY DATA
