# Supplementary material for: Formation of circular polyribosomes on eukaryotic mRNA without cap-structure and poly(A)-tail: a cryo electron tomography study
Source: Nucleic Acids Res. 2014 Jul 12;42(14):9461–9. doi: 10.1093/nar/gku599 (PMC4132722; doi:10.1093/nar/gku599)
Supplement: SUPPLEMENTARY DATA [file supp_42_14_9461__index.html]

Formation of circular polyribosomes on eukaryotic mRNA without cap-structure and poly(A)-tail: a cryo electron tomography study — Formation of circular polyribosomes on eukaryotic mRNA without cap-structure and poly(A)-tail: a cryo electron tomography study — SUPPLEMENTARY DATA 

# Formation of circular polyribosomes on eukaryotic mRNA without cap-structure and poly(A)-tail: a cryo electron tomography study

## SUPPLEMENTARY DATA

**Files in this Data Supplement:**

- SUPPLEMENTARY DATA
