# Supplementary material for: Formation of circular polyribosomes on eukaryotic mRNA without cap-structure and poly(A)-tail: a cryo electron tomography study
Source: Nucleic Acids Res. 2014 Jul 12;42(14):9461–9. doi: 10.1093/nar/gku599 (PMC4132722; doi:10.1093/nar/gku599)
Supplement: SUPPLEMENTARY DATA [file supp_gku599_nar-03602-h-2013-File003.pdf]

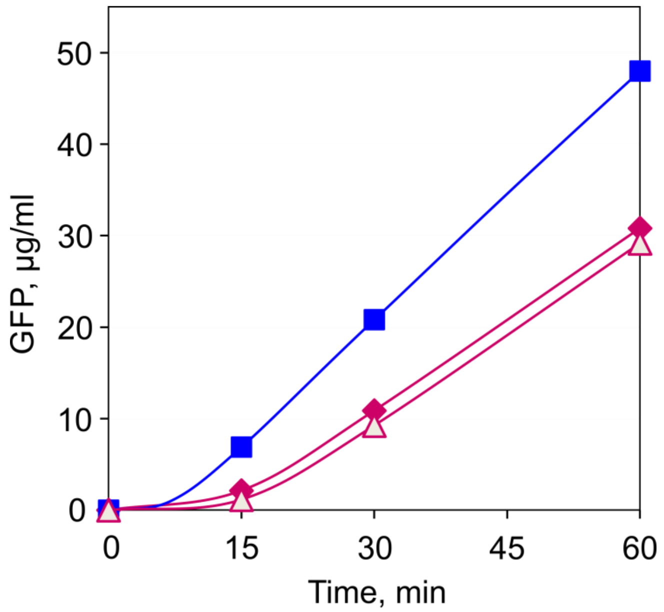

Figure S1. Time course of translation in wheat germ cell-free system with optimal concentrations of mRNAs: 100 nM *Cap-5'UTR*$_{\beta Globin}$*-scGFP-3'UTR*$_{(N)40\text{-}(A)100}$ (◆), 200 nM *Cap-5'UTR*$_{\beta Globin}$*-scGFP-3'UTR*$_{(N)180}$ (△) and 300 nM *5'UTR*$_{Obe}$*-GFP-3'UTR*$_{TMV}$ (■). The reaction was performed as described in Materials and Methods. Samples for cryo-ET and sedimentation analysis were taken at 15 min of incubation.

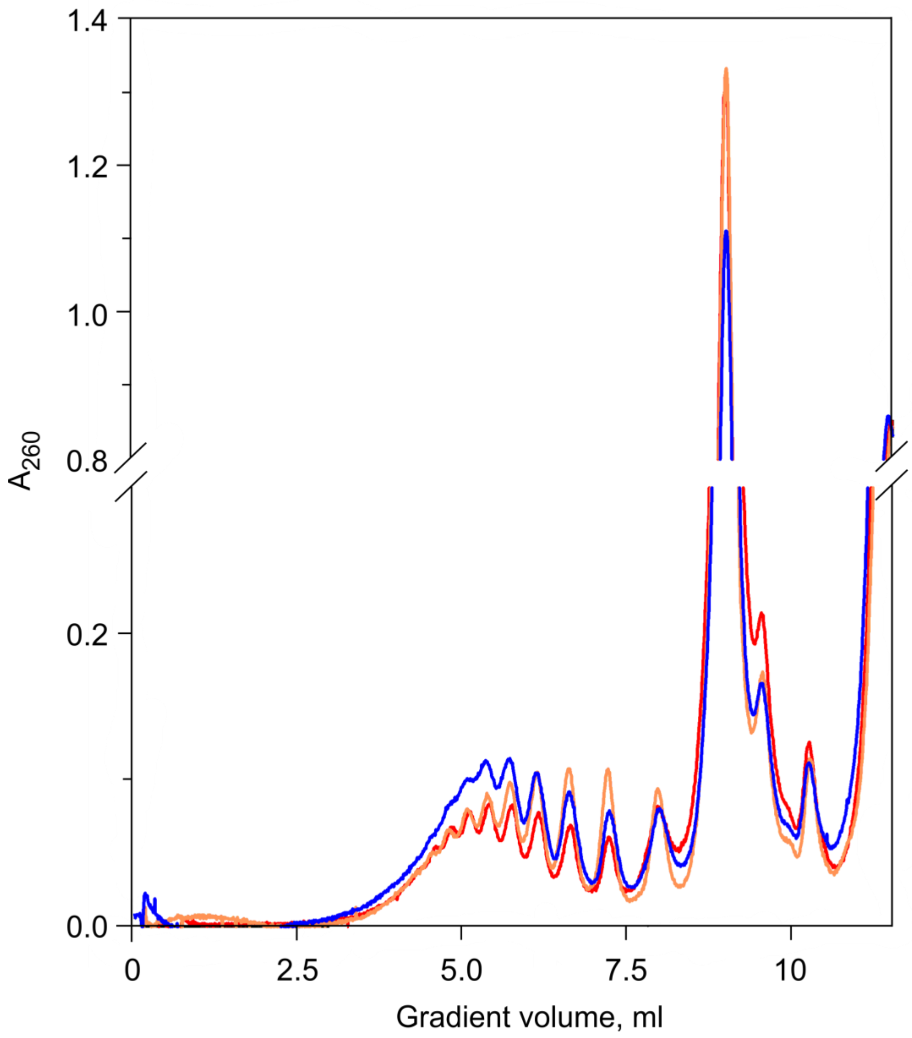

Figure S2. Sedimentation analysis of polyribosome profile in translation mixtures after 15 min of incubation (samples from reactions shown in Figure S1). 25 μl of translation mixture were stopped by addition of 75 μl cold buffer A containing 5 mM $Mg(OAc)_2$ and 0.01 mg/ml cycloheximide, and analyzed in 15-50% linear sucrose gradient in the same buffer by centrifugation at 37000 rpm for 2h at 4C in SW-41 rotor. Translation was directed by capped mRNA with poly(A) tail *Cap-5'UTR*$_{\beta Globin}$*-scGFP-3'UTR*$_{(N)40-(A)100}$ (red), capped mRNA without poly(A) tail *Cap-5'UTR*$_{\beta Globin}$*-scGFP-3'UTR*$_{(N)180}$, (orange), or uncapped non-polyadenylated mRNA *5'UTR*$_{Obe}$*-GFP-3'UTR*$_{TMV}$ (blue).

Table S1. Occurrence of circular and linear polyribosomes in the cryo-ET specimens.

	$Cap\text{-}5'UTR_{\beta Globin}\text{-}scGFP\text{-}3'UTR_{(N)40\text{-}(A)100}$			$Cap\text{-}5'UTR_{\beta Globin}\text{-}scGFP\text{-}3'UTR_{(N)180}$			$5'UTR_{Obe}\text{-}GFP\text{-}3'UTR_{TMV}$		
	6 tomograms			5 tomograms			4 tomograms		
**Polyribosome type**	total #	**circular**	**linear**	total #	**circular**	**linear**	total #	**circular**	**linear**
# of polyribosomes	162	77	85	128	51	77	134	70	64
% of all polyribosomes		**48**	52		**40**	60		**52**	48
# of polysomal ribosomes	874	352	522	637	233	404	821	360	461
% of all polysomal ribosomes		**40**	60		**37**	63		**44**	56

Polyribosomes in the reconstructed tomograms were classified circular or non-circular according to the criteria based on the topology of the inferred mRNA path, as described in Methods and Results sections. Total number of polyribosomes and number of polyribosomes of circular and non-circular (linear) topology in all tomograms with particular mRNAs, as well as number of ribosomes comprising these polysomes were counted. Only tetrasomes and larger polysomes were accounted in this analysis.

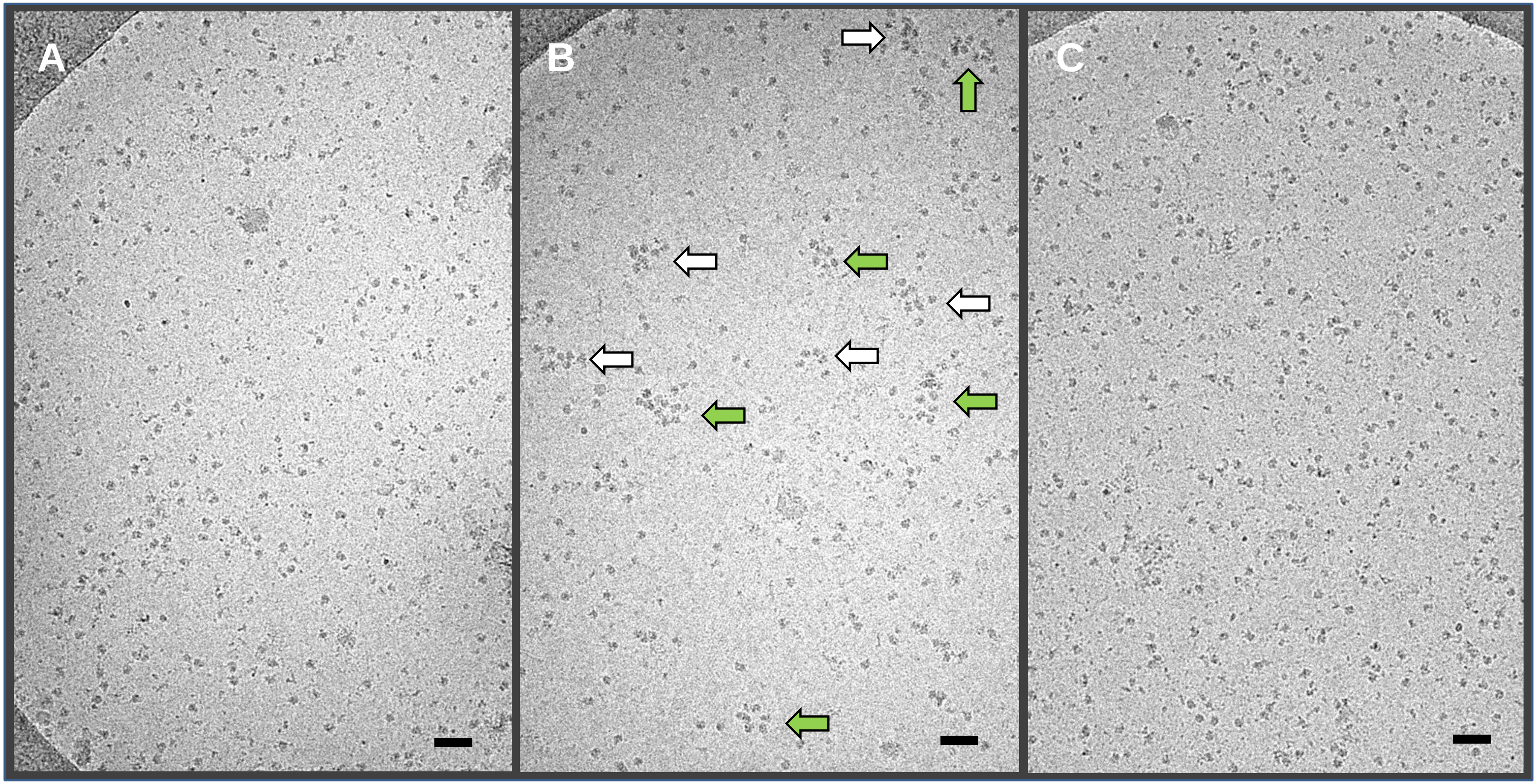

Figure S3.
mRNA dependent formation of polyribosomes in a wheat germ cell-free translation system, and the effect of puromycin. Translation mixtures prepared as described in Methods were incubated for 15 min at 25°C either without mRNA (A), or with 300 nM *5'UTR*$_{Obe}$*-GFP-3'UTR*$_{TMV}$ mRNA (B and C). In (C) 1 mM puromycin was added after translation and the sample was further incubated for 15 min at 25°C. Samples were diluted 5-fold with buffer A prepared as described in Methods and visualized by cryo-EM (Polara F30, 100kV, 15000x). Arrows in B point to polyribosomes, green arrows indicate presumably circular polyribosomes. Scale bar 100 nm.

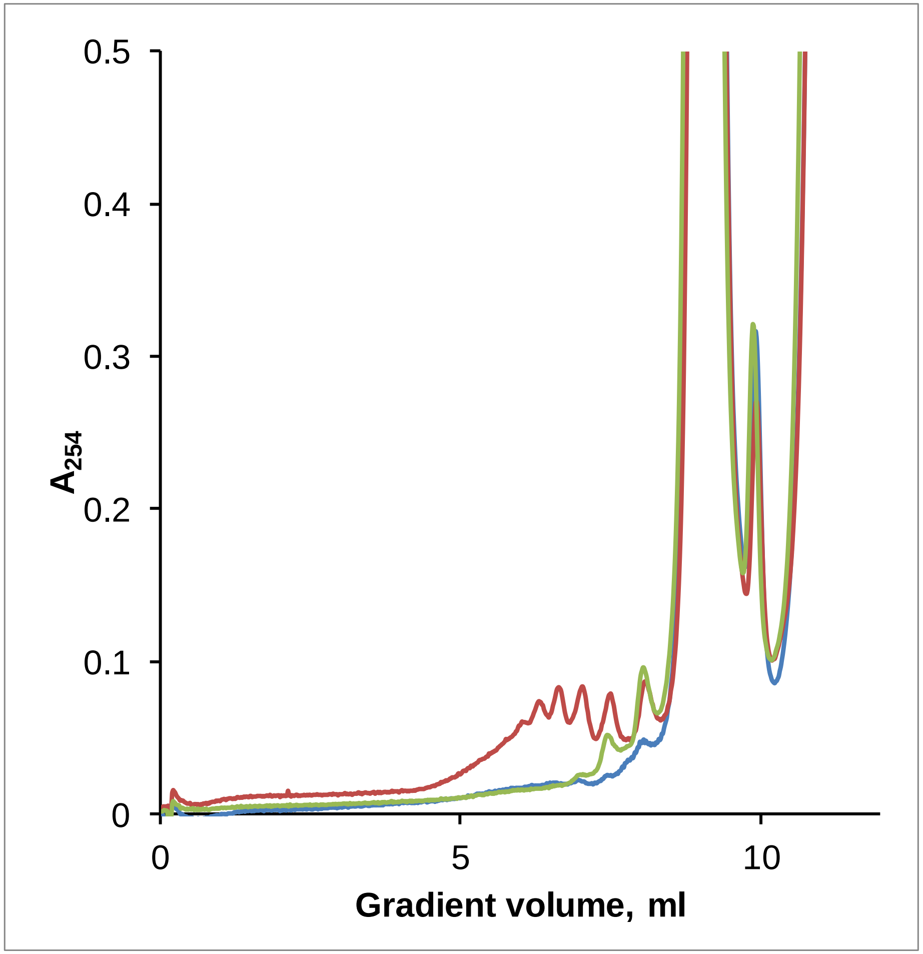

Figure S4.
Sedimentation analysis of polyribosomes formed in wheat germ cell-free translation system and puromycin test. Translation mixtures were incubated for 15 min at 25°C without mRNA (blue line) or with 300 nM *5'UTR*$_{Obe}$-*GFP-3'UTR*$_{TMV}$ mRNA (red and green lines). In puromycin test (green line) the sample after translation was supplemented with 1 mM puromycin and further incubated for 15 min at 25°C. Samples were quenched with 0.1 mg/ml CHX. 20 µl aliquots of the reaction mixture were loaded on 15-50% sucrose gradient in 25 mM HEPES-KOH pH7.6 buffer with 1.5 mM $Mg(OAc)_2$, 85 mM KOAc, 0.01 mg/ml of cycloheximide. After 2 hrs centrifugation at 37000 rpm in SW41 rotor the gradients were pumped out with monitoring UV-absorbance (A_{254}) and fluorescence (Ex 495 nm/Em 515 nm, green line) in flow cells as described in (30).

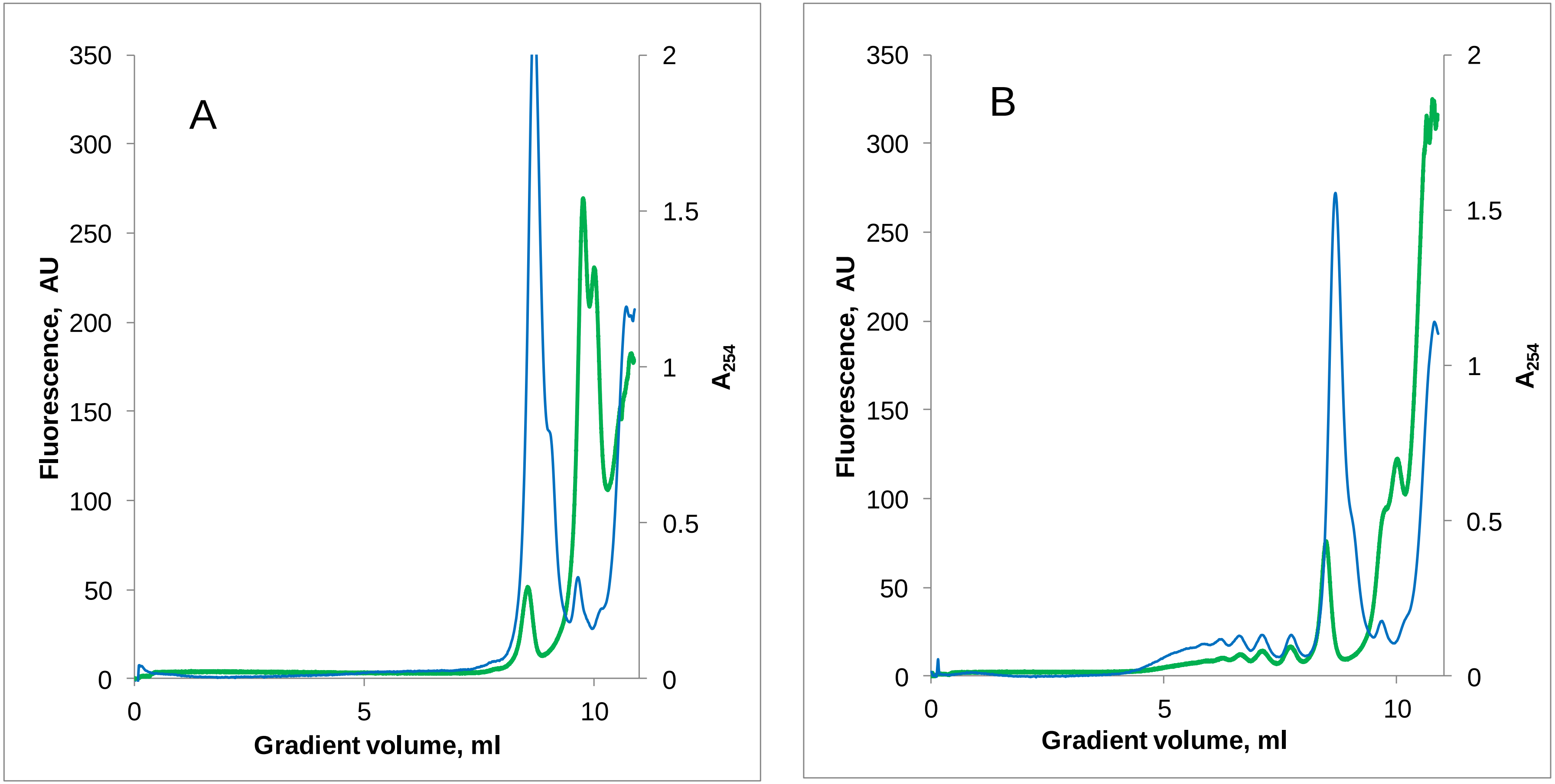

Figure S5.

Sedimentation analysis of the wheat germ cell-free translation system with 500 nM *5'UTR*$_{Obe}$*-GFP-3'UTR*$_{TMV}$ mRNA 3'-labeled with fluorescein demonstrates incorporation of mRNA in polyribosomes. A, translation reaction was quenched with CHX immediately upon addition of mRNA ; B, translation reaction proceeded for 15 minutes at 25°C then quenched with CHX. 20 µl aliquots of the reaction mixture were loaded on 15-50% sucrose gradient in 25 mM HEPES-KOH pH7.6 buffer with 1.5 mM Mg(OAc)$_2$, 85 mM KOAc, 0.01 mg/ml of cycloheximide. After 2 hrs centrifugation at 37000 rpm in SW41 rotor the gradients were pumped out with monitoring UV-absorbance (A_{254} , blue line) and fluorescence (Ex 495 nm/Em 515 nm, green line) in flow cells as described in (30). The UV/Fluorescence ratio decreases in heavier polysomes in correspondence with increasing the ribosome/mRNA ratio.

Figures S6-S11
Movie files (mpeg1) with reconstructed polyribosome models shown in Figs. 2B-4B.
Movies generated in UCSF Chimera software.

Figs. S6 and S7 (complementary to Fig. 2B).
Reconstructed models of polyribosomes formed on capped mRNA with poly(A) tail
$\mathit{Cap\text{-}5'UTR}_{\boldsymbol{\beta Globin}}\mathit{\text{-}scGFP\text{-}3'UTR}_{\boldsymbol{(N)40\text{-}(A)100}}$
Files: <cap-polyA#10>
<cap-polyA#28>

Figs. S8 and S9 (complementary to Fig. 3B).
Reconstructed models of polyribosomes formed on capped mRNA without poly(A) tail
$\mathit{Cap\text{-}5'UTR}_{\boldsymbol{\beta Globin}}\mathit{\text{-}scGFP\text{-}3'UTR}_{\boldsymbol{(N)180}}$
Files: <cap-nonpolyA#14>
<cap-nonpolyA#19>

Figs. S10 and S11 (complementary to Fig. 4B).
Reconstructed models of polyribosomes formed on uncapped non-polyadenylated mRNA
$\mathit{5'UTR}_{\boldsymbol{Obe}}\mathit{\text{-}GFP\text{-}3'UTR}_{\boldsymbol{TMV}}$
Files: <uncap-nonpolyA#19>
<uncap-nonpolyA#22>
